# Miller Fisher Syndrome as a Stroke Mimic: A Case Report

**DOI:** 10.7759/cureus.79997

**Published:** 2025-03-03

**Authors:** Anmol Multani, Miguel A Leon, Lorlelei Lee-Haynes, Edward J Durant

**Affiliations:** 1 Department of Emergency Medicine, Kaiser Permanente, Modesto, USA; 2 Department of Internal Medicine, Cleveland Clinic Foundation, Cleveland, USA; 3 Department of Graduate Medical Education, Kaiser Permanente, Modesto, USA

**Keywords:** acute stroke mimic, ataxia, deep tendon areflexia, guillan-barre syndrome, miller fisher syndrome, peripheral neuropathy

## Abstract

Guillain-Barre syndrome (GBS) is a heterogeneous autoimmune disorder characterized by peripheral neuropathy, often triggered by preceding infections or vaccinations. It encompasses several clinical variants, including the rare Miller Fisher Syndrome (MFS), distinguished by ophthalmoplegia, ataxia, and areflexia. Diagnosis is challenging due to varied presentations and potential overlap with other neurological conditions. We present a case of a 42-year-old male initially suspected to have a stroke when he presented with unilateral loss of sensation and dysarthria. He was later diagnosed with MFS after his condition progressed and he developed generalized weakness, ophthalmoplegia, ataxia, and areflexia. Despite initial stability, his condition deteriorated, requiring intensive care. Early recognition and treatment, such as intravenous immunoglobulin (IVIg) and plasmapheresis, are critical for improving outcomes in GBS and its variants. This case underscores the importance of clinical suspicion and appropriate diagnostic strategies in managing these complex neurological disorders.

## Introduction

Guillain-Barre syndrome (GBS) is an often misdiagnosed condition of peripheral neuropathy that results in some degree of permanent disability in up to 20% of patients and is fatal in around 5-10% of patients [1]. One of the challenges in diagnosing GBS stems from its varied clinical presentation. The clinical spectrum of GBS is divided into two major groups: acute inflammatory demyelinating polyradiculoneuropathy (AIDP) and the axonal subtypes, including axonal motor neuropathy (AMAN) and acute sensory axonal neuropathy (ASAN) [2]. The diagnostic criteria for GBS, defined by the United States National Institute of Neurological Disorders and Stroke (NINDS) and the Brighton Collaboration Guillain-Barre Syndrome Working Group, include bilateral flaccid weakness of proximal and distal limbs and decreased or absent deep tendon reflexes [2]. It is caused by an autoimmune attack on the peripheral nerve gangliosides, usually after a viral or bacterial infection via molecular mimicry [1,2]. Although GBS is a reversible condition with a favorable prognosis and spontaneous resolution in most patients, it can cause neurological dysfunction that results in 20% of patients being unable to ambulate without any assistance one year from disease onset and death in 5% of patients [1]. During the acute phase of the condition, 10-30% of patients may end up needing mechanical ventilation due to respiratory complications [2]. In many cases, patients report respiratory tract or gastrointestinal tract infections prior to the onset of the symptoms of GBS [1]. Many viral and bacterial infections have been implicated in triggering this autoimmune response, including Epstein-Barr virus, Cytomegalovirus, human immunodeficiency virus, *Haemophilus influenzae*, *Mycoplasma pneumoniae*, influenza A, and Zika virus [2], but *Campylobacter jejuni* (*C. jejuni*) bacterial infection is the most common [1]. The development of GBS is not limited only to infections; it has also been linked to certain vaccines [3].

Miller Fisher syndrome (MFS) was identified in 1956 as one of the rare variants of GBS [4]. It is a kind of AIDP that is characterized by a triad of ophthalmoplegia, ataxia, and areflexia, and patients must present with at least one of these clinical findings to be diagnosed with MFS [4]. The incidence of GBS is 1 in 100,000 [5]. MFS is rarer, with an incidence of 1 in 1,000,000 [5]. The difficulty in diagnosing MFS arises from the overlap in symptoms between MFS and typical GBS presentation, as they both involve peripheral neuropathy [6]. Both conditions also have cerebrospinal fluid (CSF) albumin-cytological dissociation or elevated CSF protein without pleocytosis [6]. MFS can also be confused with other life-threatening neurological conditions such as brainstem stroke, drug intoxication, myasthenia gravis, and botulism, which can delay treatment [7]. Here, we present a case of a patient who initially presented to the emergency department (ED) with symptoms consistent with stroke and was later found to have MFS.

## Case presentation

**Figure 1:**
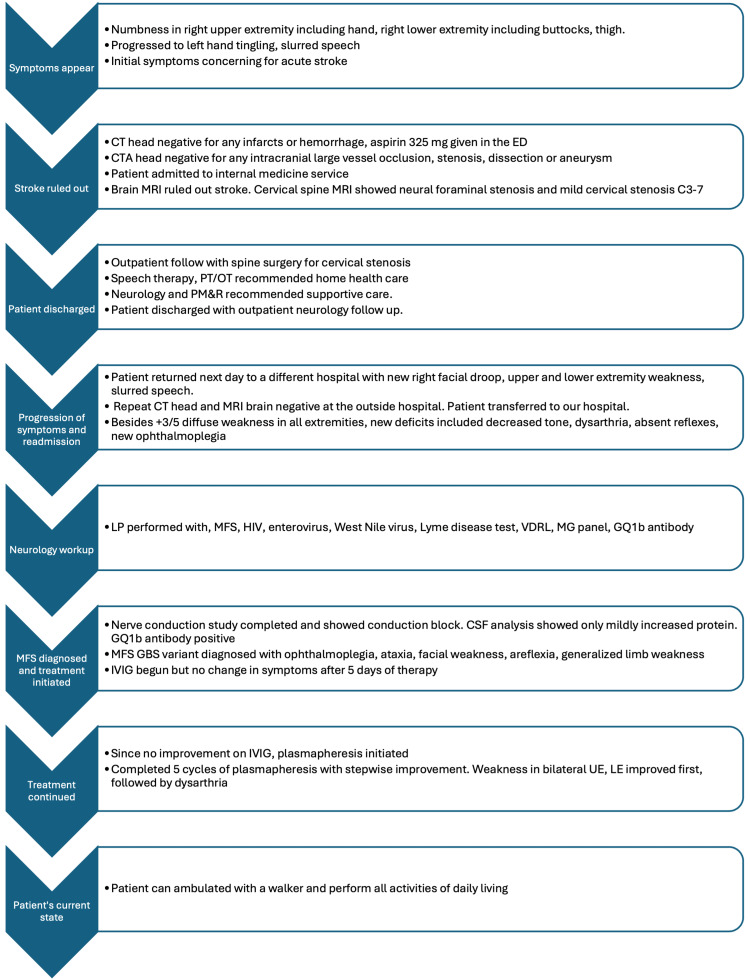
Timeline of the major events in the case CT: computed tomography; CTA: computed tomography angiogram; ED: emergency department; MRI: magnetic resonance imaging; PT/OT: physical therapy and occupational therapy; PM&R: physical medicine and rehabilitation; LP: lumbar puncture; HIV: human immunodeficiency virus; VDRL: Venereal Disease Research Laboratory levels; MG: myasthenia gravis; IVIG: intravenous immunoglobulin; GBS: Guillain-Barré syndrome; MFS: Miller Fisher syndrome; CSF: cerebrospinal fluid

The patient was a 42-year-old male with no significant past medical history who presented to the ED with sudden onset numbness in his right hand, right buttocks, and thigh and slurring of speech. His symptoms started at 02:00, and he presented to the ED at 22:13 when his symptoms did not improve. He was not within the tissue plasminogen activator (tPA) window for acute stroke and did not receive any tPA. Two hours prior to coming to the ED, he also developed a tingling sensation in his left hand. He reported having an upper respiratory tract infection and a few episodes of diarrhea in the weeks prior to his ED presentation, for which he treated himself with two doses of ivermectin. The timeline of the case is illustrated in Figure 1.

On physical examination, he had right arm drift, right-sided decreased sensation, and slurred speech with an NIHSS score of 3. He also exhibited right-sided finger-to-nose dysmetria, but his reflexes were intact. Computed tomography (CT) without contrast of the head was completed at 23:23, which did not reveal any acute infarcts or hemorrhage. The patient was not hypertensive (BP < 130/80); therefore, no antihypertensive treatment was given. He was given atorvastatin 80 mg along with aspirin 325 mg as secondary stroke prevention. CT angiography of the head and neck was also obtained, and it did not show large vessel occlusion, stenosis, or intracranial abnormalities. He was not found to have heart arrhythmias like atrial fibrillation, and there was no evidence of thrombi on the transthoracic echocardiogram.

The patient was admitted to the internal medicine service. The electrocardiogram did not have any abnormalities, and the complete blood count, complete metabolic panel, and troponin level were all within normal limits. Magnetic resonance imaging (MRI) of the brain ruled out stroke. An MRI of the cervical spine was completed as the patient complained of tingling in the fingertips. It showed neural foraminal stenosis and mild cervical stenosis C3-7. Because the patient was medically stable, he was discharged with outpatient neurology follow-up, physical therapy, and occupational therapy at home.

The patient did not have any worsening neurological deficits or decline in functional status. Neurology evaluated the patient and recommended outpatient follow-up with spine surgery for cervical stenosis. Acute stroke was ruled out, and no clear etiology was found for dysarthria and lack of sensation. Speech therapy, physical therapy, and occupational therapy are recommended for home health care. The physical medicine and rehabilitation team evaluated the patient and recommended supportive care with neurology. The patient was then discharged with outpatient neurology follow-up.

After being discharged, the patient returned the next day to a different hospital with a progression of symptoms, including worsening right facial droop, right upper and lower extremity weakness, and slurred speech. CT head without contrast and MRI of the brain were repeated at the outside hospital and were found to show no abnormalities. The patient was then transferred to our hospital. When he was examined neurologically, he had +3/5 diffuse weakness in all extremities, decreased tone, absent reflexes, ophthalmoplegia, and ataxic gait, which were all new findings. During this readmission, the on-call neurologist recommended a lumbar puncture. Additional labs were completed, including CSF protein, glucose, cell count, gram stain (Table 1), venereal disease research laboratory levels (VDRL), myasthenia gravis panel, and viral panel including human immunodeficiency virus, enterovirus, and West Nile virus.

**Table 1:** Cerebrospinal fluid analysis results CSF: cerebrospinal fluid; WBC: white cell count

CSF test	Analysis results	Reference range and units
Glucose	66	50-80 mg/dL
Protein	48	15-45 mg/dL
WBC	5	≤5/mcL
Color	Colorless	Colorless
Clarity	Clear	Clear

CSF analysis showed only mildly increased protein, and the viral panel and all other tests were negative. Autoantibodies against the ganglioside complex GQ1b were detected in the CSF, and the patient’s stool cultures grew *C. jejuni*. The neurologist suspected the Miller Fisher variant of GBS given the presence of ophthalmoplegia, ataxia, and areflexia. In order to treat it, intravenous immunoglobulin (IVIg) treatments were initiated at the dose of 400 mg/kg for five days. Additionally, a nerve conduction study was also performed, which showed conduction block and dropped F waves. The motor and sensory nerves had decreased amplitude, which was also consistent with the electromyography results showing reduced amplitude of contraction in all muscle groups. Despite completing five days of IVIg, the patient’s condition did not improve. His diffuse muscle weakness also affected the muscles involved in respiratory function, and he was subsequently intubated because he developed acute hypoxic respiratory failure with an altered mental status and inability to maintain adequate oxygen saturation despite non-invasive oxygen support.

Because of the lack of improvement with IVIg treatment, plasmapheresis was started on the patient, which resulted in significant clinical improvement. A total of five cycles of plasmapheresis were completed with stepwise improvement with each cycle. He was extubated, and his weakness in bilateral upper and lower extremities improved. Dysarthria improved at a slower rate. The patient was then discharged, and an electromyography was completed outpatient to track improvement, which showed recovery of muscle strength. As of this writing, two years after the event, the patient can ambulate with a walker and perform his activities of daily living.

## Discussion

The patient’s initial presentation was concerning for stroke because of the unilateral loss of sensation and dysarthria. In contrast, GBS usually presents as a symmetric weakness in the upper or lower extremities. GBS is characterized by an acute immune response damaging nerves and nerve roots and mediating polyradiculopathies, which leads to progressive symmetrical limb weakness and absent or decreased deep tendon reflexes [8,9]. In some cases, GBS can also present as weakness primarily affecting the cervical, pharyngeal, and brachial muscles [10]. Neurologic deficits typically seen in MFS are also different from what we observed initially in our patient because MFS usually begins with a craniocaudal pattern of weakness, beginning with ophthalmoplegia [11]. Instead, our patient initially developed predominantly unilateral loss of sensation with subsequent progression to ophthalmoplegia, ataxia, generalized muscle weakness, and areflexia, which is how MFS symptomology is classically described. Other conditions that can cause an acute onset of ataxia also need to be ruled out, such as cerebellar lesions [11]. Consumption of toxins like alcohol and medications like phenytoin and chemotherapeutic agents, which alter sodium levels, can also lead to an acute onset of ataxia [11]. The symptoms typically reported to be associated with a misdiagnosis of GBS are intact deep tendon reflexes and neuropathic pain, of which our patient had the former [12]. As GBS progressed in our patient, more symptoms emerged, and others became more prominent. Muscle weakness leading to respiratory failure emerged, and subsequently, the ophthalmoplegia, ataxia, and areflexia became more obvious.

In cases with confounding symptoms, confirmatory tests like serological markers can be used to accurately diagnose. Anti-GQ1b antibodies are strongly associated with MFS and were positive in our patient, supporting the diagnosis of MFS [11]. In most cases, GBS is preceded by an upper respiratory or gastrointestinal illness, as was the case with our patient’s preceding upper respiratory infection and with the isolation of *C. jejuni* from stool.

GBS is a disease with varying presentations, including AMAN, AIDP, ASAN, MFS, and pharyngeal cervical brachial variants [13]. Some uncommon presentations that are reported in the literature include unilateral limb weakness, unilateral or bilateral facial palsy, pharyngeal weakness, sensory disturbance with ataxia, dysautonomia, or bulbar predominant weakness [9,14]. One study also found 11 cases of patients having predominantly bulbar weakness with initial slurred speech, ataxia, diplopia, and dizziness [15]. Some patients were also found to have paraparesis restricted to legs with no upper extremity involvement in the initial presentation of GBS or paraparesis with intact reflexes [14,16,17]. Paraparesis is also found to be the first symptom to appear prior to weakness [16]. Overall, GBS has variable presentations and symptom severity, which are reported in the literature. We shared a presentation of GBS in our patient's case that is dissimilar to the clinical presentations reported previously in this area. Our patient initially developed loss of sensation unilaterally, dysarthria without weakness, and with intact reflexes. Areflexia and bilateral weakness in the extremities emerged later. His condition deteriorated, leading to ophthalmoplegia, along with ataxia and dysarthria. Our patient had symptoms consistent with two different GBS variants: AIDP, where both sensory and motor changes occur, and MFS, where a triad of ophthalmoplegia, ataxia, and areflexia is observed.

It is important to make a timely diagnosis of GBS despite its heterogeneous nature because GBS can become a life-threatening condition, causing dysrhythmias, labile blood pressure, respiratory failure, and bulbar palsy [18]. It took more than one evaluation before a GBS diagnosis was confirmed for 65% of patients because of its heterogeneous presentations [13]. Early IVIg or plasma exchange treatments within two weeks of symptom onset can prevent disease progression [9]. IVIg was initially used in our case because it has traditionally been the preferred treatment due to its ease of use compared to plasmapheresis. There is insufficient evidence to demonstrate the superiority of IVIg or plasmapheresis therapy over the other [19]. Our patient did not respond to initial treatment with IVIg. For patients who do not respond to IVIg, there is currently insufficient evidence to guide treatment in GBS. Plasmapheresis is considered an alternative. However, the results of studies are mixed for outcomes between those receiving a second cycle of IVIg and those treated with plasmapheresis [20]. Our case demonstrated positive outcomes to plasmapheresis after our patient failed to respond to IVIg with improvement in neurological deficits, including weakness. Recovery in GBS patients can take weeks to months; however, treatment with plasmapheresis or IVIg can hasten the recovery process [6]. Our patient had stepwise improvement in weakness in all muscle groups within one week of beginning plasmapheresis.

Early supportive management and monitoring of respiratory status can improve patient prognosis [12,13]. Without initial evaluation and treatment, complications like residual weakness and respiratory distress become more likely [9]. With high clinical suspicion, confirmatory tests can be conducted, including electrodiagnostic findings like abnormal F wave, prolongation of onset latency, and slowing of conduction velocity [18]. Additionally, CSF analysis is supportive of GBS diagnosis when CSF protein >45 mg/dL (normal limit: 15-40 mg/dL) and WBC <95/dL (normal limit: 0-5/dL) [13,17]. However, 10% of GBS patients may have normal CSF studies [5].

## Conclusions

GBS and its rare variant, MFS, present significant challenges in diagnosis due to their diverse clinical manifestations. Our patient’s case highlights the importance of recognizing atypical presentations of the GBS spectrum of autoimmune conditions, as the patient’s initial symptoms mimicked symptoms from a stroke. Despite the rarity of MFS, clinicians should maintain a high index of suspicion, especially in cases with neurological deficits involving ophthalmoplegia, ataxia, and areflexia. Confirmatory tests such as neurological markers and electrodiagnostic studies can help accurately diagnose GBS and its variants, ensuring prompt initiation of appropriate management like IVIg or plasma exchange.
